# An Optimized Clustering Routing Algorithm for Wireless Sensor Networks Based on Spotted Hyena and Improved Energy-Efficient Non-Uniform Clustering

**DOI:** 10.3390/s26092866

**Published:** 2026-05-03

**Authors:** Songhao Jia, Shuya Jia, Wenqian Shao, Fangfang Li

**Affiliations:** School of Artificial Intelligence, Nanyang Normal University, Nanyang 473061, China; jsonghao@nynu.edu.cn (S.J.); shaowenqian@nynu.edu.cn (W.S.); liff@nynu.edu.cn (F.L.)

**Keywords:** Wireless Sensor Networks (WSNs), swarm optimization algorithm, cluster head selection, energy consumption, network lifetime

## Abstract

Wireless Sensor Networks (WSNs) are widely used in environmental monitoring, disaster early warning, and smart grids. However, sensor nodes face strict energy limitations. Unbalanced energy consumption and hotspots severely shorten the network lifetime. To address these problems, this paper proposes an optimized Spotted Hyena Optimization-Energy-Efficient Non-Uniform Clustering algorithm (SHOE) for cluster head selection and data transmission. The algorithm has three main innovations: combining a bio-inspired metaheuristic with an improved EEUC (Energy-Efficient Unequal Clustering) multi-hop relay and a Gaussian distribution model for non-uniform node deployment; designing a multi-dimensional fitness function considering energy, distance, and node location; and introducing empty cluster and isolated node repair mechanisms to balance exploration and exploitation. Specifically, the multi-dimensional fitness function guides the heuristic search process towards high-quality cluster head candidates, while the empty cluster and isolated node repair mechanisms dynamically rectify abnormal network structures, ensuring the robustness of the final architecture optimized by the bio-inspired framework. Simulations in MATLAB show that SHOE outperforms LEACH (Low-Energy Adaptive Clustering Hierarchy), PSOE (Particle Swarm Optimization with Evolutionary Strategy), PL-EBC (Probabilistic Localized Energy-Balanced Clustering), and CGWOA (Chaotic Grey Wolf Optimization Algorithm) in reducing node death, saving energy, and extending network lifetime. It improves adaptability to non-uniform distribution and optimizes energy balance, thus enhancing the efficiency and stability of WSNs.

## 1. Introduction

Wireless Sensor Networks (WSNs) serve as the core sensing units of the Internet of Things (IoT) technology system and have integrated deeply into numerous critical scenarios such as intelligent security, marine monitoring, geological disaster warning, smart grid operation, and cold chain logistics tracking. The typical characteristic shared by these application scenarios is that sensor nodes often need to be arranged in harsh environments, complex terrains, and areas lacking human maintenance conditions [1]. For instance, in deep-sea ecological monitoring, the nodes need to withstand long-term tests in high-pressure, low-temperature, and highly corrosive environments; in mountainous geological disaster warning, the nodes need to be rooted in steep slopes and rocky areas, and endure extreme natural events such as heavy rain, mountain floods, and earthquakes; in remote areas of smart grid monitoring, the nodes need to be distributed in vast deserts and along high-voltage lines, facing complex conditions with significant temperature differences between day and night, and high risks of lightning strikes.

**Figure 1:**
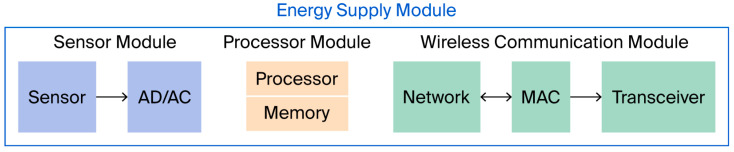
Structure Diagram of Sensor Node.

In these application scenarios, the core mission of sensor nodes is to achieve the continuous perception of physical quantities (for example, vibration, temperature and humidity, voltage and current, gas concentration, etc.), data preprocessing, and remote transmission. The core resources driving these core functions are the limited energy built into the nodes. Due to the miniaturized design of the nodes, cost control during deployment, and outdoor installation requirements, sensor nodes typically have limited battery capacity. The battery structure of the sensor nodes is shown in Figure 1. The need to replace the batteries manually involves crossing terrain obstacles, dealing with harsh environments, and not only poses extremely high operational difficulty and time cost but also may cause accidental damage to the monitoring area’s ecological environment or key infrastructure; what is more concerning is that in some high-risk scenarios (such as nuclear radiation monitoring, toxic gas leakage warning), the presence of humans near the nodes poses serious safety hazards, and battery replacement is simply not feasible [2]. Additionally, due to the actual monitoring areas often being constrained by terrain, obstacles, signal attenuation, and deployment costs, the nodes cannot achieve ideal uniform coverage. This non-uniform distribution directly affects the fairness of cluster head election, the reliability of routing paths, and the balance of energy consumption. Therefore, introducing the simulation constraints of uneven node distribution during the algorithm design stage can more accurately simulate the topological characteristics and communication bottlenecks in actual deployments, thereby improving the adaptability and robustness of the protocol in the real environment.

More importantly, the energy consumption of sensor nodes has the characteristics of “non-renewable” and “differential”: once the battery energy is exhausted, the node will permanently lose its sensing and transmission capabilities. If the nodes in key areas fail in concentration, they will cause a “data gap” in the monitoring network, thereby leading to the paralysis of the entire system; and this differential characteristic is reflected in the role division of the nodes. Cluster head nodes need to undertake additional tasks such as data aggregation and cross-cluster forwarding, and their energy consumption rate is much higher than that of ordinary nodes only responsible for data collection. Especially the cluster head nodes near the base station need to undertake a large amount of cross-cluster data forwarding tasks, which are prone to form “energy hotspots”, accelerating their own failure and triggering network segmentation.

In wireless sensor networks for the Internet of Things, node mobility, adaptive communication protocols, and clustering mechanisms jointly determine energy efficiency and network stability [3,4,5]. Mobility introduces dynamic topological changes and intermittent links, while routing and MAC protocols directly affect competition, retransmission, and relay loads. Recent research has confirmed that clustering efficiency, neighbor discovery mechanisms, and energy-aware routing are crucial for reducing hotspots and extending network lifetime in heterogeneous and mobile wireless sensor networks. Zhuwaied et al. emphasize that neighbor-aware clustering can significantly improve stability in heterogeneous deployments; Garzanano et al. point out that chat-based routing methods can enhance network stability and reduce the cost of dynamic topological structures; Rajaram et al. demonstrated that the energy optimization-based clustering protocol can effectively balance the load and extend the operation time. These studies collectively indicate that energy conservation in wireless sensor networks is not determined by a single mechanism, but rather by the collaborative effect of deployment, mobility, clustering, and routing. Therefore, how to achieve the balanced distribution of network energy load during operation is an urgent problem to be solved.

## 2. Critical Review of Existing Approaches

### 2.1. Current Research Status at Home and Abroad

Cluster-based routing protocols for wireless sensor networks are key technologies for achieving efficient energy transmission and load balancing in the network. With the rapid development of the Internet of Things and 5G applications, WSN is facing challenges such as limited node energy, complex data aggregation, and dynamic topology changes. Clustering routing significantly reduces energy consumption and prolongs network lifetime by grouping nodes into clusters and selecting cluster heads for data fusion and forwarding to the base station. In recent years, the research focus has shifted from classical probability models to intelligent optimization algorithms, such as PSOE [6] and PL-EBC [7] based on particle swarm optimization, CGWOA [8] based on grey wolf optimization, and the classic LEACH protocol [9]. These algorithms aim to optimize cluster head selection, inter-cluster routing, and energy balance to meet the requirements of heterogeneous networks and large-scale deployments.

In recent years, domestic and international researchers have placed greater emphasis on algorithm innovation and theoretical framework construction. The classic LEACH protocol serves as the foundation of clustering routing, achieving simple load balancing through the random probability selection of cluster heads, but it has problems of uneven cluster head distribution and energy hotspots. Therefore, researchers have introduced meta-heuristic algorithms for improvement. For example, algorithms based on PSO, such as PSOE, use the particle swarm optimization algorithm as the core, addressing the disorderly distribution of LEACH cluster heads, by dividing the network into quadrants and optimizing the cluster head positions in each region, reducing the communication distance within clusters, but the algorithm only focuses on “minimizing the intra-cluster communication distance”, without considering the transmission energy consumption from cluster heads to the base station, resulting in the cluster heads in the closer-to-the-base station quadrant becoming energy hotspots due to excessive forwarding pressure, while the edge quadrant cluster heads have idle energy consumption, ignoring the global energy balance, and achieving low global energy utilization [10]. PL-EBC further integrates PSO with energy thresholds, selects candidate cluster heads and optimizes the total number of cluster heads to “total network energy consumption (intra-cluster + inter-cluster)”. As the fitness function, it optimizes the total number and positions of cluster heads in the candidate cluster head set through PSO iterations, balancing the load of each region’s cluster heads. At the same time, a simple multi-hop mechanism is introduced for edge cluster heads, prioritizing the selection of intermediate cluster heads with sufficient remaining energy as relays, reducing long-distance single-hop transmission energy consumption, and improving multi-hop routing efficiency [11]. However, multi-hop routing only uses “remaining energy” as the relay selection criterion, without quantifying the energy difference between “direct transmission” and “relay selection”, making it prone to choosing the more energy-intensive ultimate path. Meanwhile, the dual-layer optimization of candidate cluster heads + PSO global iteration significantly increases the algorithm running time and is not suitable for large-scale WSN [12]. Inspired by the grey wolf algorithm, CGWOA simulates the hunting behavior of wolves, achieving the joint optimization of cluster heads and routing through the update of α, β, δ, and ω positions, adapting to uneven node distribution and dynamic convergence factor A and random factor C to achieve an “exploration–development” adaptive balance [13]. Meanwhile, the algorithm in this paper integrates the improved EEUC for the energy difference criterion of multi-hop relays, significantly reducing long-distance transmission energy consumption and effectively solving the problems of global imbalance, poor distribution adaptation, and the insufficient convergence of existing algorithms [14,15] (Table 1).

### 2.2. Algorithm Introduction

#### 2.2.1. Spotted Hyena Optimization Algorithm

The Spotted Hyena Optimization Algorithm (SHOA) is a swarm intelligence optimization algorithm that simulates the hunting behavior of a spotted hyena group. Its inspiration comes from the natural behavior of spotted hyenas, namely “hierarchical collaboration—surrounding the prey—dynamic attack”. This process guides the population direction through four social levels: α, β, δ, and ω. Combined with the dynamic adjustment of convergence factors and random factors, it achieves a precise balance of “global exploration and local exploitation”. In mathematical modeling, SHOA uses distance calculation, position update formulas, and fitness iteration to efficiently locate the global optimal solution in a high-dimensional and multi-constraint search space, with both convergence speed and optimization accuracy.

In wireless sensor networks, the selection of cluster heads requires a comprehensive consideration of multiple dimensions such as energy consumption, spatial distribution, and communication delay, which is a typical multi-objective NP-hard combinatorial optimization problem [15]. The SHOA algorithm, with its hierarchical mechanism for strong guidance of the global optimal solution, the adaptability of the fitness function (see formula (1)) to multiple objectives, the characteristics of few parameters and low computational cost, and its good robustness in uneven node distribution scenarios, becomes an ideal solution for cluster head selection in wireless sensor network clustering routing. Compared to the random clustering of the traditional LEACH protocol and the premature convergence problem of the PSO algorithm, SHOA not only significantly improves the uniformity of cluster head distribution but also reduces the total network energy consumption through full-link energy optimization, effectively alleviating the “energy hotspot” problem and significantly extending the network lifetime. In specific implementation, the “population initialization + empty cluster / isolated point repair + dynamic threshold constraint” approach is adopted to ensure a reasonable number of cluster heads (5–10) that comply with the clustering principle of EEUC “close near and far apart”. The initial population is generated by combining “Gaussian distribution initialization and energy threshold filtering”, which is both suitable for the characteristics of uneven node distribution in actual scenarios and avoids the premature failure of low-energy nodes being selected as cluster heads.

(1) $$f=w_{1}\cdot E_{\mathrm{total}}+w_{2}\cdot \mathrm{avgNode}2\mathrm{CH}+w_{3}\cdot \mathrm{Var}\left(E_{\mathrm{CH}}\right)-w_{4}\cdot \mathrm{avgCH}2\mathrm{BS}$$

$$\begin{array}{cc} E_{total} & =\frac{E_{A}-E_{min}}{E_{max}-E_{min}}\\ avgNode2CH & =\frac{{avgNode2CH}_{max}-avgNode2CH}{{avgNode2CH}_{max}-{avgNode2CH}_{min}}\\ Var(E_{CH}) & =\frac{Var(E_{CH}{)}_{max}-Var(E_{CH})}{Var(E_{CH}{)}_{max}-Var(E_{CH}{)}_{min}}\\ avgCH2BS & =\frac{{avgCH2BS}_{max}-avgCH2BS}{{avgCH2BS}_{max}-{avgCH2BS}_{min}} \end{array}$$

Among them, $E_{\mathrm{total}}$ represents the overall energy dissipation of the network avgNode2CH is the average distance from an ordinary node to the cluster head, $\mathrm{Var}\left(E_{\mathrm{CH}}\right)$ is the variance of the remaining energy of the cluster head, avgCH2BS is the average distance from the cluster head to the base station, and $w_{1}$–$w_{4}$ are the weight coefficients, satisfying $\;\sum_{i=1}^{4}w_{i}=1$.

#### 2.2.2. EEUC Multi-Hop Relay Selection Mechanism

The EEUC multi-hop relay selection strategy is a clustering routing optimization mechanism designed to address the energy hotspot issue in WSN. Its core idea stems from the communication logic of “non-uniform clustering + energy-optimal relay” [16]. By dynamically adjusting the cluster coverage radius and multi-hop paths, it achieves a “close-near distant” cluster structure and global energy balance. In communication modeling, EEUC completes the intelligent switch between “direct transmission” and “multi-hop transmission” through distance threshold judgment, energy difference calculation, and relay node selection, effectively balancing the energy consumption caused by long-distance transmission and the load of relay forwarding.

In wireless sensor networks, the single-hop long-distance transmission from the cluster head to the base station is the core cause of “energy hotspots”, as the cluster head close to the base station needs to undertake a large amount of forwarding tasks, which may die prematurely leading to network segmentation [17]. The essence of relay selection is a multi-constraint optimization problem, requiring the simultaneous consideration of factors such as transmission distance, remaining node energy, and total path energy consumption. The EEUC multi-hop relay selection strategy, with its characteristics of “non-uniform clustering adaptive topology”, “energy difference quantification of path superiority”, and “dynamic threshold adaptation to network status”, can precisely match the communication requirements of WSN: it reduces the forwarding pressure by shrinking the coverage radius of the cluster close to the base station, and decomposes the long-distance high-energy consumption into short-distance low-energy consumption through multi-hop relaying. Compared with traditional single-hop transmission or fixed multi-hop strategies, it has significant advantages in energy balance and network lifetime. In specific implementation, the “distance threshold judgment + cost energy difference calculation + relay node priority sorting” method is adopted to ensure the energy optimality of the relay path: When the distance from the cluster head to the base station exceeds the threshold, the energy difference between “direct transmission” and “relay transmission” is calculated using formula (2), and the cluster head with sufficient remaining energy closer to the base station is selected as the relay to avoid invalid forwarding; at the same time, the coverage radius of the cluster close to the base station is dynamically reduced (e.g., linearly decreasing from 90 m to 30 m), ensuring the sufficient number of cluster heads in the near-base station area, and further distributing the forwarding load [18].

(2) $$Cost=E_{CH\to Relay}+E_{Relay\to BS}-E_{CH\to BS}$$

## 3. Wireless Sensor Network Configuration

### 3.1. System Model

(1) Monitoring Area and Node Deployment

In this paper’s simulation experiments, the monitoring area is modeled as a 2D rectangular region of 800 m × 800 m, with node coordinates strictly constrained within the defined boundaries.

(2) Node Types

There are three types of nodes in the area, and the initial energy is uniformly set to E0 = 3J:

Sensor Nodes: There are n = 100 of them, randomly and unevenly deployed in the monitoring area, responsible for data collection and transmission;

Cluster Head Nodes: They are dynamically elected by the SHOE algorithm from the sensor nodes, responsible for data aggregation within the cluster and data forwarding between clusters;

Base Station: It is fixedly deployed at the geometric center of the monitoring area, responsible for receiving and processing the data of the entire network, with unlimited energy.

### 3.2. Node Deployment Characteristics

The distribution characteristics of nodes are one of the core foundations of the wireless sensor network model, directly determining the energy consumption optimization direction of the clustering algorithm and the operational efficiency of the network. In this paper, the sensor nodes of the SHOE algorithm are deployed unevenly by Gaussian distribution, with the core focusing on the principle of “central concentration and boundary constraint” [19].

To more realistically reflect the spatial heterogeneity in the actual monitoring scenario, this paper adopts a non-uniform node deployment strategy based on the Gaussian distribution of the SHOE algorithm. Different from uniform random deployment, the Gaussian distribution enables nodes to achieve a controllable concentrated distribution around the monitoring core while maintaining global coverage. The complete process of node generation is as follows:

Firstly, the monitoring area is defined as a two-dimensional square region Ω = [0, xm] × [0, ym], where xm = 800 m and ym = 800 m. The base station (BS) is deployed at the geometric center of this area, that is, (xBS, yBS) = (xm/2, ym/2) = (400, 400). To ensure that the node density is the highest near the base station and gradually decreases towards the boundary, the node coordinates are independently generated along the x-axis and y-axis using the Gaussian distribution:

xi∼N(μx, σ2), yi∼N(μy, σ2)

where

$$\begin{array}{c} \mu x=x_{m}/2=400,\;\mu y=y_{m}/2=400\\ \sigma =x_{m}/4=200 \end{array}$$

This configuration ensures that the statistical center of node deployment coincides exactly with the location of the base station, thereby forming a radiating pattern of decreasing node density.

The deployment process of each sensor node i can be described as follows:

1. Random sampling

Generate two independent Gaussian random variables:

$$x_{i}=\mu_{x}+\sigma \cdot randn(),\;y_{i}=\mu_{y}+\sigma \cdot randn(),$$

where randn() represents a standard normal random variable.

2. Boundary constraint handling

Since the Gaussian distribution is theoretically unbounded, some sampling points may exceed the monitoring area. To ensure that all nodes are within Ω, a boundary handling mechanism is adopted:

xi=min(max(xi,0),xm), yi=min(max(yi, 0), ym)

For example, a resampling strategy can also be used, that is, discard the samples that exceed the range and regenerate them until an effective result is obtained.

3. Node set construction

Repeat the above steps until all n sensor nodes are generated:

S=(x1,y1),(x2,y2),…,(xn,yn)

From a probabilistic perspective, the Gaussian distribution conforms to the classic “3σ rule”: approximately 68% of the nodes fall within: [μ−σ, μ + σ] ⇒ [200, 600]; approximately 95% of the nodes fall within: [μ−2σ, μ + 2σ] ⇒ [0, 800].

This means most nodes are concentrated in the central area, forming a high-density cluster, while a small number of nodes naturally extend towards the network boundary, ensuring the integrity of coverage. This distribution forms a “dense center, sparse edge” topological structure, which is highly consistent with the actual deployment mode. Gaussian deployment can promote higher node density near the base station for the SHOE algorithm, which naturally results in smaller cluster sizes near the central area and larger cluster sizes in the edge area, thereby reducing the “hotspot” phenomenon; secondly, the high-density central nodes make the selection of cluster heads more flexible, thus avoiding the premature exhaustion of energy in key nodes; at the same time, edge nodes can use the dense intermediate nodes as relay points, significantly reducing energy consumption for long-distance transmission and improving multi-hop efficiency, better reflecting the actual deployment scenario [20].

**Figure 2:**
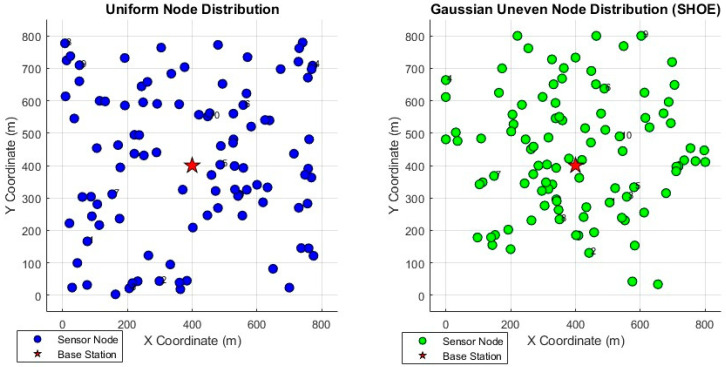
Comparison of uniform distribution of nodes and Gaussian non-uniform distribution.

In this study, σ = 200 m was selected as an empirical optimal value, which achieved a balance between node concentration and spatial coverage, thus ensuring efficient energy utilization and the robustness of the network. Figure 2 is a comparison diagram of uniform node distribution and Gaussian non-uniform distribution scenarios:

### 3.3. Energy Consumption Modelling

This study utilizes the typical energy consumption model for wireless communication. Energy consumption is calculated in three categories: “transmission, reception, and aggregation”. The parameters are uniform and fixed. The core energy consumption parameters are shown in the Table 2:

This paper adopts the classic first-order radio energy consumption model to calculate the energy consumption for both data transmission and reception by the nodes. The energy consumed for transmitting k bits of data is:

(3) $$E_{Tx}\left(k,d\right)=\left\{\begin{array}{ll} k\cdot E_{elec}+k\cdot E_{fs}\cdot d^{2} & \mathrm{if}\;d<d_{0}\\ k\cdot E_{elec}+k\cdot E_{mp}\cdot d^{4} & \mathrm{if}\;d\geq d_{0} \end{array}\right.$$

When the transmission distance d between two communicating nodes exceeds the threshold d_0_, the multipath fading channel model is adopted. Conversely, when the distance d is less than d_0_, the free space channel model is utilized.

Here, Eelec denotes the energy consumption of the node’s circuitry per bit (unit: J/bit), Efs stands for the attenuation coefficient of the free space channel (unit: J/(bit·m^2^)), Emp represents the attenuation coefficient of the multipath fading channel (unit: J/(bit·m^4^)), and d0 is defined as the transmission distance threshold between nodes. d_0_ = $\sqrt{\frac{E_{fs}}{E_{mp}}}$. The energy consumption of the node for receiving one bit of data is only related to the circuit operation, and the formula is: $E_{rx}(l)=E_{elec}\times l$.

### 3.4. Algorithm Design

#### 3.4.1. Cluster Head Selection Strategy Based on Spot Hyena Algorithm

The essence of the Spotted Hyena Optimization algorithm lies in striking a balance between global exploration and local exploitation via the iterative mechanism of hierarchical ranking, prey encircling, and hunting behavior, so as to obtain the optimal solution for optimization problems [21]. It can be applied to the cluster head selection mechanism in the clustering routing of wireless sensor networks during the process of finding the optimal solution. Therefore, this paper uses this algorithm to elect cluster heads and transforms the process of the spot hyena finding prey into the process of selecting the optimal cluster head in wireless sensor networks.

(1) Initial Population Setup

In the initial population setup stage of the algorithm, pop candidate cluster head combinations are generated, and each combination corresponds to the “position” of the hyena. This step is the starting point of the algorithm and corresponds to the assembly stage of the spot hyena group before hunting.

The quality of population diversity in the initial stage directly affects the global search capability of the algorithm. In this paper, the value of pop is set to 20–50 to balance the computational efficiency and search range [22]. The number of nodes in each candidate combination is dynamically determined by the cluster head probability chp to ensure that the initial solution meets the actual requirements of WSN clustering. To avoid invalid solutions, initialization ensures that each combination has nodes that are alive and have sufficient energy.

(2) Iterative Optimization

(a) Calculate the fitness value of each candidate combination based on energy consumption

For each candidate cluster head combination, calculate the total energy consumption of the entire network. The calculation formula is as follows:

(4) $$\mathrm{Fitness}=\sum_{i=1}^{N}E_{\mathrm{send}}\left(i\to {CH}_{i}\right)+\sum_{j=1}^{K}E_{\mathrm{recv}}\left({CH}_{j}\right)+\sum_{j=1}^{K}E_{\mathrm{send}}\left({CH}_{j}\to BS\right)$$

Among them, $E_{\mathrm{send}}\left(i\to {CH}_{i}\right)$ represents the energy consumption when an ordinary node sends data to its cluster head; $E_{\mathrm{recv}}\left({CH}_{j}\right)$ represents the energy consumption when the cluster head receives and aggregates the data; $E_{\mathrm{send}}\left({CH}_{j}\to BS\right)$ represents the energy consumption when the cluster head sends data to the base station.

The smaller the fitness value is, the lower the energy consumption of the cluster head combination, and the closer to the “optimal solution”, that is, the position of the prey.

(b) Simulate the “rank guidance” of the spotted hyena and divide them into α, β, δ, and ω levels.

Sort all the candidate combinations in step (1) in ascending order of fitness values and select the first three groups with the smallest fitness values as the α (optimal), β (second-best), and δ (third-best) hyenas, representing the individuals closest to the “prey” (optimal solution). The remaining candidate groups will be regarded as ω (ordinary) hyenas, and they will follow the directions of α, β, and δ.

This step simulates the social hierarchy of spotted hyenas and is the key to the efficient convergence of the algorithm. Through hierarchical ranking, the population is always guided by the current optimal individual, avoiding blind searching and significantly improving convergence efficiency [23].

(c) Balance global exploration and local exploitation, and update positions

This step simulates the process of hyenas “surrounding the prey–attacking the prey” through the dynamic adjustment of candidate combinations, which is the core mathematical model of the algorithm. For each ω hyena, calculate the distances to its α, β, and δ, and the calculation formula is as follows:

(5) $$D_{\alpha }=\left|C_{1}\cdot X_{\alpha }-X_{1}\right|,\;D_{\beta }=\left|C_{2}\cdot X_{\beta }-X_{1}\right|,\;D_{\delta }=\left|C_{3}\cdot X_{\delta }-X_{1}\right|$$

Based on the distance and convergence factor A, three guiding directions are generated:

(6) $$X_{1}=X_{\alpha }-A_{1}\cdot D_{\alpha },\;X_{2}=X_{\beta }-A_{2}\cdot D_{\beta },\;X_{3}=X_{\delta }-A_{3}\cdot D_{\delta }$$

The new position is obtained by averaging the three guiding directions:

$$x_{i}^{t+1}=\frac{x_{i-1}+x_{i}+x_{i+1}}{3}$$

Among them, random factor C takes values between 0 and 2 to simulate the random movement behavior of hyenas, allowing individuals to have a probability of escaping from local optima and avoiding the algorithm from falling into local optimal solutions thus ensuring the robustness of the algorithm. Convergence factor A decreases linearly from 2 to 0, controlling the search range. When |A| > 1, the algorithm conducts global exploration, quickly traversing the solution space and moving away from the current optimal solution, searching new areas; when |A| < 1, the algorithm conducts local development, conducting a fine search near the optimal solution and approaching the current optimal solution.

(3) Determine the Optimal Solution

After the iteration process is completed, the global optimal solution (α Hyena) recorded in all iterations is the final optimal cluster head combination. The nodes in this combination will be marked as cluster heads and used for subsequent clustering and data transmission [24].

**Figure 3:**
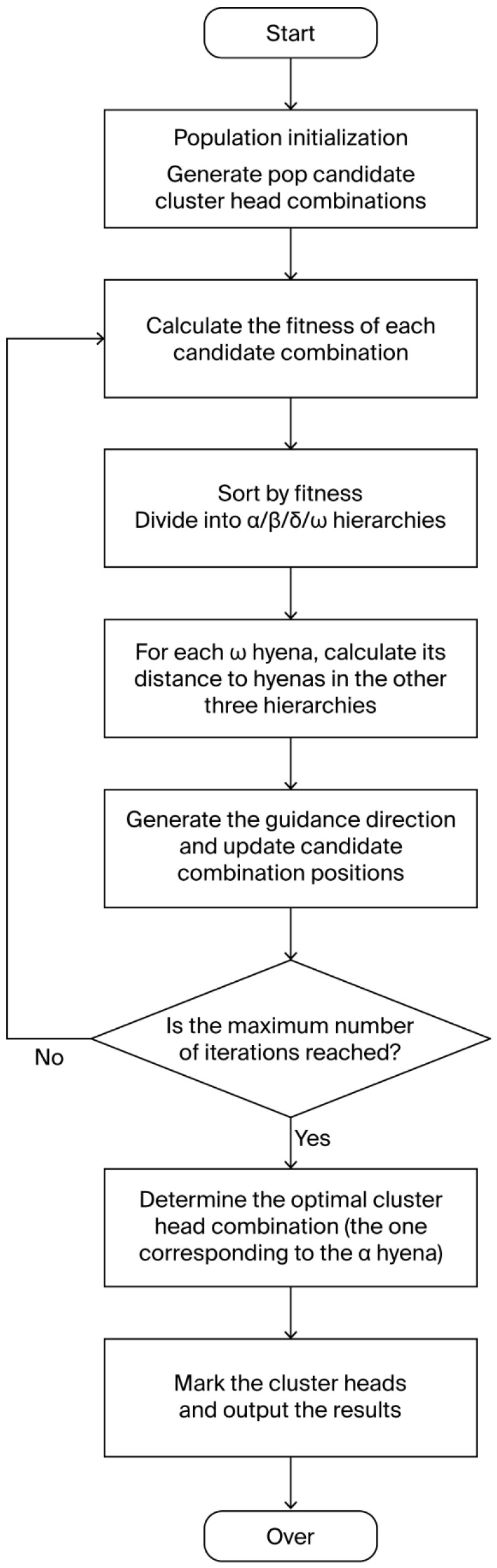
Flowchart of Cluster Head Selection Algorithm.

The flowchart of the proposed cluster head selection strategy using the spotted hyena algorithm is presented Figure 3.

#### 3.4.2. Empty Clusters, Isolated Points Repair and Dynamic Clustering

(1) Repair Strategies for Empty Clusters and Isolated Points

In the optimization process of SHOA, problems such as “empty clusters” (clusters without ordinary nodes as cluster heads) and “isolated points” (where the distance between a normal node and its nearest cluster head is too far) may occur. The algorithm incorporates a robust repair mechanism:

As for empty clusters, if a cluster head has no member nodes associated with it, this node will be reclassified as a regular node to prevent unnecessary energy dissipation. For isolated node processing, the distance from each ordinary node to its nearest cluster head is computed [25]. If this distance exceeds the empirical threshold of 50 m, the corresponding node is promoted to a new cluster head, and the cluster head set and positions are updated accordingly. This mechanism guarantees that every node can access its closest cluster head, which is consistent with the “near-to-close, far-to-distant” clustering rule employed in the EEUC algorithm.

(2) Dynamic Clustering

In each round of the network cycle, the clustering procedure and node status are dynamically synchronized. First, the number of surviving nodes and overall energy consumption are counted, and the node coordinates are updated. Then, the cluster head selection and repair mechanisms of SHOE are performed to determine the final cluster heads. Regular nodes associate with their closest cluster head according to the minimum distance principle, thereby completing the cluster structure establishment and laying a foundation for subsequent data transmission.

#### 3.4.3. Multi-Hop Routing Transmission Strategy Based on Relay Selection

(1) Cluster Internal Communication

Cluster internal communication adopts a single-hop mode of “ordinary node → cluster head”, the core of which is to reduce energy consumption through distance optimization.

Ordinary nodes receive the clustering information broadcast by the cluster head, consuming the receiving energy:

(7) $$E_{RX}\times L_{ctr}=50\times 10^{-9}\times 100=5\times 10^{-6}\;J,\;L_{ctr}=100\;bit$$

Each ordinary node sends its data packets to the corresponding cluster head, and the energy consumption of such transmission is computed based on the distance-dependent energy consumption model. The cluster head is responsible for receiving the data packets and executing data fusion, where the energy consumption for data reception and aggregation can be expressed as:

(8) $$\left(E_{RX}+E_{DA}\right)\times L=\left(50+5\right)\times 10^{-9}\times 2000=1.1\times 10^{-4}\;J$$

The cluster head distribution selected through SHOA ensures that the average distance from ordinary nodes to the cluster heads is minimized, thereby avoiding the high energy consumption caused by long-distance intra-cluster transmission.

(2) Improved EEUC Algorithm

This paper adopts the improved EEUC multi-hop mechanism to optimize the transmission path from “cluster head → base station”.

Selection of the original EEUC relay nodes:

The cluster head first determines the distance dtoBS to the base station. If dtoBS < TD_MAX = 150 m, it directly transmits; otherwise, it calls the CostEEUC function to select the optimal relay node. The CostEEUC function selects the relay node by calculating the energy consumption difference between “direct transmission energy consumption” and “relay transmission energy consumption”. The calculation formula is as follows:

(9) $$Cost=\left(E_{CH\to Relay}+E_{Relay\to BS}\right)-E_{CH\to BS}$$

If Cost < 0, it indicates that relay transmission is more energy-efficient. Therefore, the cluster head with sufficient remaining energy and closer distance to the base station should be selected as the relay node. Otherwise, the data is directly transmitted to the base station.

Once relay transmission is chosen, the source cluster head sends data packets to the relay cluster head, consuming transmission energy. The relay cluster head receives and caches the aggregated data packets. The relay cluster head will repeat these two steps continuously until the data packet is transmitted to a cluster head within a distance less than TD_MAX from the base station. Finally, this cluster head transmits the data to the base station.

The TD_MAX in the original EEUC algorithm is a fixed value, which makes it unsuitable when the network density or energy changes. Long-distance nodes may perform excessive multi-hop or directly transmit, wasting energy. SHOA, based on EEUC, dynamically calculates TD_MAX:

(10) $$TD\text{\_}MAX\text{\_}dynamic=150\cdot \left(1+node\text{\_}density\cdot 2\right)\cdot avg\text{\_}energy\text{\_}ratio$$

Thereinto, $\mathrm{node}\text{\_}\mathrm{density}=\frac{\mathrm{length}\left(alive\text{\_}nodes\right)}{xm\times ym},\;\mathrm{avg}\text{\_}\mathrm{energy}\text{\_}\mathrm{ratio}=\frac{\mathrm{mean}\left(\left[SH\left(alive\text{\_}nodes\right).E\right]\right)}{Eo}$.

Secondly, implement dynamic limit on the number of hops: use a while loop to ensure that the number of hops does not exceed max_pop.

Meanwhile, a primary–backup relay selection and switching mechanism is introduced to enhance the robustness and energy balance of inter-cluster communication. In the original EEUC algorithm, only a single relay node is selected for data forwarding, and no redundant relay path is provided. Although such a strategy is simple to implement, it suffers from poor adaptability under dynamic network conditions. Once the selected relay node experiences rapid residual energy depletion, temporary overload, or communication degradation, the source cluster head cannot promptly reselect another forwarding node, which may lead to packet loss, transmission interruption, excessive retransmissions, or severe local energy imbalance. Consequently, the stability and lifetime of the network are negatively affected.

To overcome this limitation, the improved EEUC algorithm constructs a relay candidate set for each transmitting cluster head and evaluates all feasible candidates using a comprehensive weighting metric [26]. The weighting function jointly considers residual energy, communication distance, relay load, and forwarding cost, thereby ensuring that the selected relay node is both energy-efficient and topologically favorable. After ranking candidate nodes according to their weights, the node with the highest score is assigned as the primary relay, while the node with the second-highest score is designated as the backup relay. This dual-relay strategy introduces redundancy into the routing process without requiring excessive control overhead.

During network operation, the primary relay is used for normal packet forwarding. Its residual energy is continuously monitored, and when the remaining energy falls below a predefined threshold,

Emainrelay<0.1E0

where (E0) denotes the initial node energy, the forwarding task is immediately transferred to the backup relay. This threshold-based switching mechanism avoids the continued use of exhausted relay nodes and prevents premature node death in heavily loaded regions. In addition, it reduces route reconstruction frequency and improves transmission continuity.

By integrating the enhanced EEUC mechanism, SHOE achieves higher efficiency and stronger adaptability in the data transmission stage. Compared with the original EEUC scheme, the proposed strategy significantly alleviates uneven energy consumption through relay load balancing, dynamic relay replacement, and backup path support. As a result, network connectivity is better maintained, hotspot node failures are reduced, and the overall lifetime of the wireless sensor network is effectively prolonged [27].

**Figure 4:**
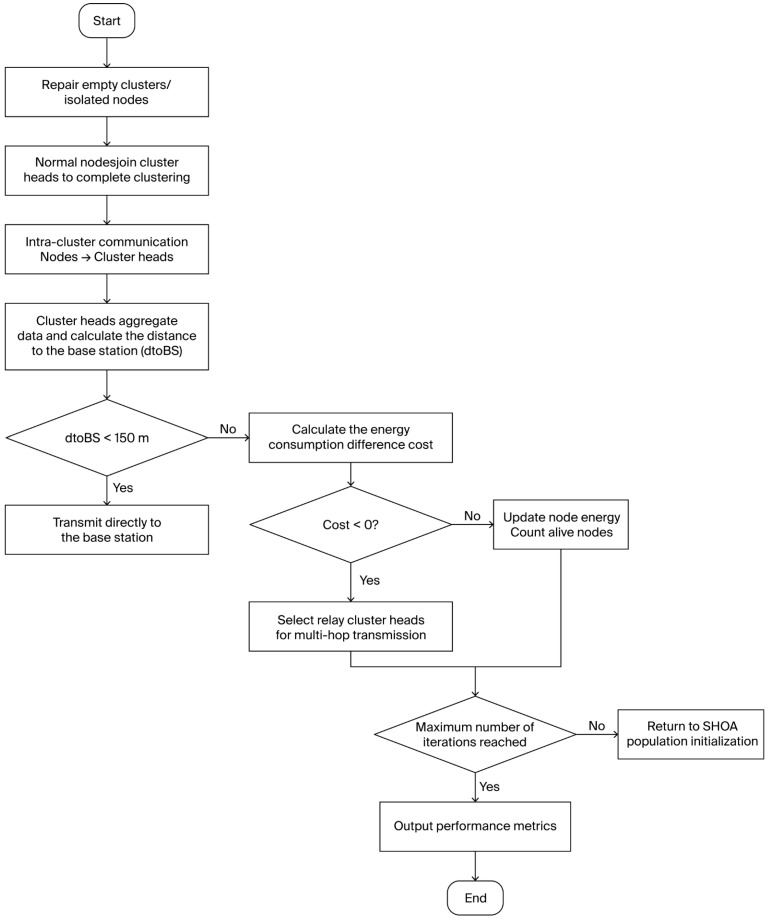
Flowchart of Data Transmission Algorithm.

The algorithm flowchart of the data transmission part is shown in Figure 4.

## 4. Algorithm Summary

This research presents a wireless sensor network (WSN) clustering routing algorithm that integrates the Spotted Hyena Optimization Algorithm (SHOA) with the Energy-Efficient Non-Uniform Clustering (EEUC) multi-hop mechanism. The core goal of this algorithm is to resolve the problems of unbalanced energy consumption and network hotspots existing in traditional clustering algorithms. The overall implementation process is divided into three interrelated modules: cluster head selection, clustering repair, and data transmission.

In the cluster head selection phase, leveraging the bionic characteristics of SHOA, 20 to 50 candidate cluster head combinations are generated through population initialization. The total energy consumption of the network is adopted as the fitness function, and hierarchical classification and position update are implemented through α/β/δ/ω levels, so as to maintain a dynamic trade-off between global exploration and the local exploitation of the algorithm [28]. The convergence factor A decreases linearly from 2 to 0, while the random factor C takes values within the interval [0, 2]. Candidate combinations are updated on the basis of distance calculation and guidance direction. After the iteration process is completed, the group corresponding to the optimal α hyena is selected as the final cluster head set.

In the clustering phase, a repair mechanism for empty clusters and isolated nodes is introduced to optimize the cluster structure: empty cluster heads (those without associated ordinary nodes) are reclassified as ordinary nodes, and isolated nodes that are over 50 m away from their closest cluster head are promoted to new cluster heads. This ensures the rationality of the cluster structure, and dynamic clustering and node status synchronization are performed simultaneously [29]. Ordinary nodes connect to the nearest cluster head in accordance with the “minimum distance principle”, which is consistent with the EEUC algorithm’s principle of “dense clustering for nearby nodes and sparse clustering for distant nodes”.

A hierarchical strategy is adopted for data transmission: within the cluster, a single-hop transmission mode is used to reduce energy consumption during short-distance communication. The energy consumption for communication between common nodes and cluster heads is determined according to the preset distance threshold. Between clusters, relay nodes are selected based on the CostEEUC function. When the condition Cost = ECH → Relay + ERelay → BS – ECH → BS < 0 is satisfied, multi-hop transmission is adopted, which decomposes long-distance high-energy consumption into short-distance low-energy consumption. This effectively avoids the premature death of hotspot cluster heads. Meanwhile, on the basis of the original EEUC algorithm, a master-slave relay selection and switching mechanism is introduced to prevent single relay failure, thus improving the network’s reliability and energy equilibrium.

Through the synergy between the optimization capability of SHOE and the energy optimization of multi-hop transmission, the algorithm proposed in this paper accomplishes dual enhancements in network lifetime and transmission efficiency, and is suitable for WSN scenarios with uneven node distribution [30].

**Figure 5:**
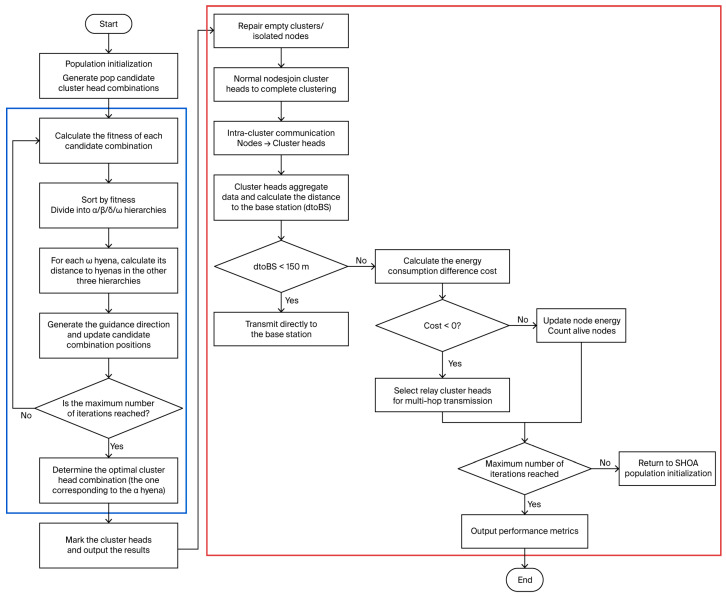
Overall Flowchart of the Algorithm.

The overall flowchart of the method adopted in this paper is shown in Figure 5 below:

## 5. Performance Simulation Analysis

For fair comparison, common network parameters and initial conditions (number of nodes, monitoring area, initial energy, radio energy consumption model, packet size, etc.) are set identically for all algorithms [31]. Meanwhile, algorithm-specific parameters of each comparative method (LEACH, PSOE, PL-EBC, CGWOA) are configured using their respective optimal values reported in the corresponding literature or widely accepted in the WSN clustering routing field [32]. Only the parameters unique to the proposed SHOE (e.g., population size, iteration count, convergence factor interval, repair threshold) adopt the values designed in this work. This setting ensures that each algorithm performs at its best while the comparison remains fair and convincing. The experiment was simulated in the MATLAB R2024a software. MATLAB R2024a is adopted because it supports the efficient implementation of the proposed metaheuristic clustering algorithm, avoids unnecessary physical-layer details involved in dedicated WSN simulators such as OMNeT++ and iFogSim, and ensures fair and consistent performance comparison with state-of-the-art algorithms in the literature.

The parameter settings of each comparison algorithm are as Table 3 follows:

All algorithms share identical initial energy, communication model, and node deployment, eliminating environmental bias.

### 5.1. Comparison of Network Dead Nodes

**Figure 6:**
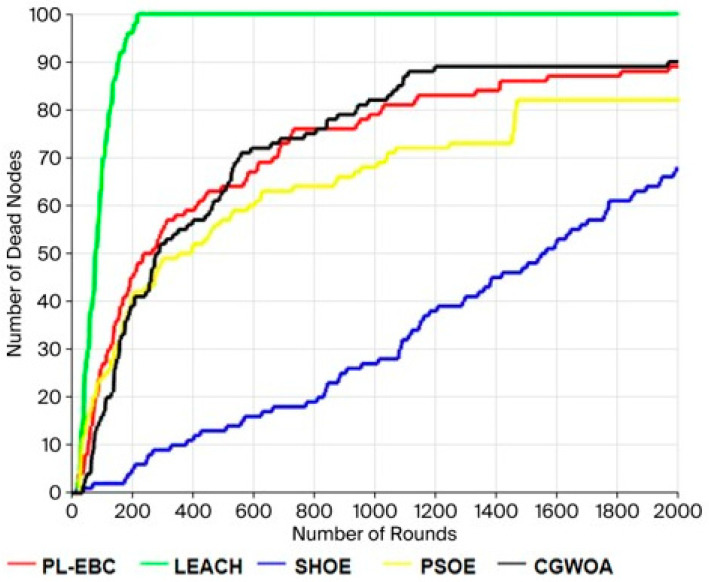
Comparison Chart of Dead Nodes Quantity.

Figure 6 illustrates the comparison of the number of dead nodes among various algorithms with the increase of iterations under identical network parameter settings. From the overall trend, it can be seen that the five algorithms have significant differences in the rate of node death and the process of network degradation, reflecting the advantages and disadvantages of their energy scheduling and routing strategies. The effectiveness of the LEACH algorithm is the weakest. The quantity of dead nodes rapidly increases to 100 around 200 iterations, indicating that the network completely fails within a very short time. This is mainly because LEACH adopts a random cluster head election mechanism, without fully considering the remaining energy of nodes and spatial distribution, resulting in some nodes prematurely undertaking high-energy-consuming tasks and rapidly depleting their energy.

In contrast, the performance of PL-EBC, PSOE, and CGWOA has improved. The growth rate of dead nodes for these algorithms is significantly slower than that of LEACH. Among them, PSOE and CGWOA have relatively concentrated node deaths in the first 300 iterations, and then the growth trend gradually slows down, indicating that these two algorithms still have certain energy distribution imbalances in the initial cluster head selection and route construction stages, but with the adjustment of the network structure, their energy consumption distribution gradually stabilizes. The overall performance of PL-EBC is between the PSO-like algorithms and LEACH. In the middle and later stages, there is still a relatively obvious cumulative node death.

It should be emphasized that the SHOE algorithm keeps the slowest growth rate of dead nodes during the entire simulation. Even at the end of 2000 iterations, the number of its dead nodes is notably lower than that of other comparative algorithms, showing superior network survival performance. This proves that SHOE can more effectively balance node energy expenditure during cluster head selection and multi-hop transmission, preventing local energy exhaustion. In general, this result fully confirms the obvious advantages of the SHOE algorithm in delaying node death and enhancing network stability.

### 5.2. Comparison of Network Surviving Nodes

**Figure 7:**
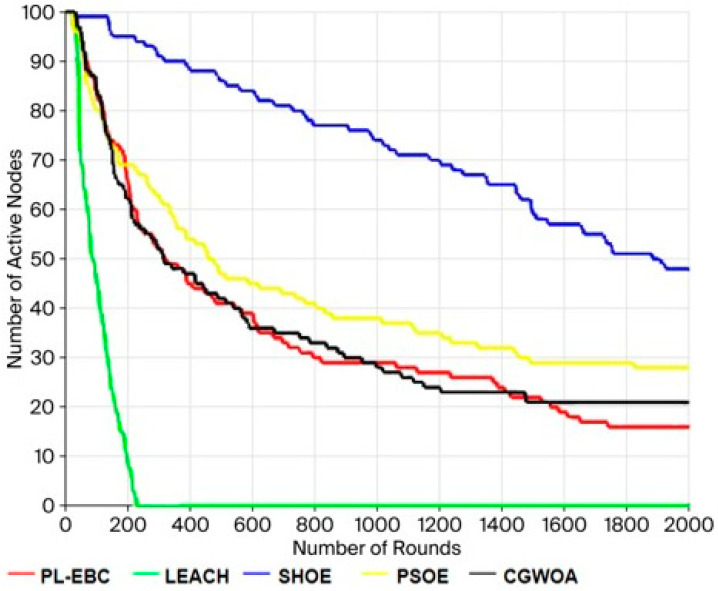
Comparison of the number of surviving nodes.

Figure 7 depicts the comparison curves of the number of surviving nodes varying with rounds during the simulation process for different algorithms. Since surviving nodes and dead nodes exhibit a complementary relationship, this indicator can more intuitively reflect the network lifecycle and overall stability. As illustrated in the figure, the number of surviving nodes of the LEACH algorithm exhibits the fastest decrease and after approximately 200 rounds, it almost drops to zero, causing the network to completely collapse, indicating that it is not suitable for application scenarios with high requirements for network lifespan.

PL-EBC, PSOE, and CGWOA algorithms show certain advantages in maintaining surviving nodes. Compared with LEACH, they can significantly extend the network’s working time. Among them, PSOE and CGWOA have a faster decline in the number of surviving nodes in the early stage, indicating that some key nodes have higher energy consumption. However, in the middle and later stages, the decline trend becomes more gradual, and the network enters a relatively stable operation stage. PL-EBC shows a relatively stable performance in the middle stage, but in the later stage, the number of surviving nodes is still lower than SHOE, indicating that its energy balance ability still has room for improvement.

The SHOE algorithm preserves the maximum number of surviving nodes during the entire simulation. Its curve declines the most slowly, showing significant network robustness. Even at the end of 2000 rounds, SHOE still retains about half of the nodes in a surviving state, while the surviving node numbers of other algorithms have dropped to a relatively low level. This indicates that SHOE can effectively avoid energy concentration consumption during cluster head election, routing adjustment, and load distribution, allowing more nodes to participate in long-term data transmission. Thus, SHOE has obvious advantages in extending the network lifecycle and improving node survival rate and is suitable for wireless sensor network scenarios with high requirements for reliability and continuous operation capabilities.

### 5.3. Comparison of Network Residual Energy

**Figure 8:**
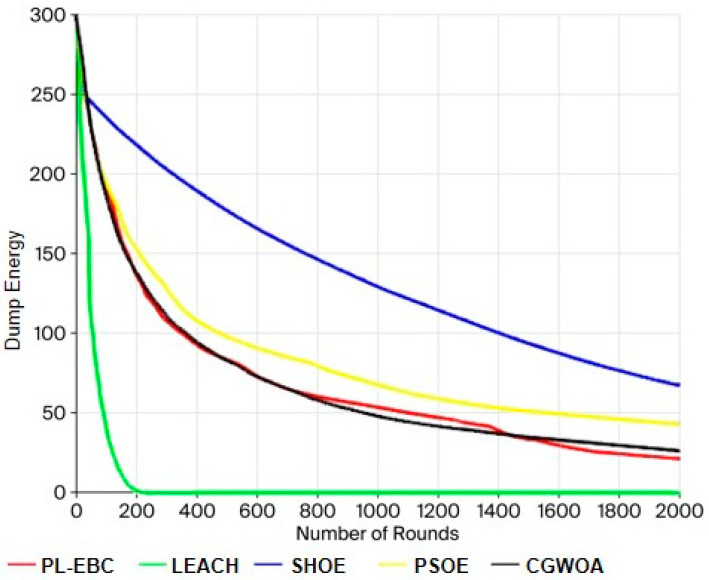
Comparison Chart of Network Remaining Energy.

Figure 8 depicts the total network residual energy of the five algorithms changing with rounds during the simulation process. It can be observed that the residual energy of all algorithms exhibits a declining trend over time, but the energy consumption rates of different algorithms are significantly different. The LEACH algorithm has the fastest energy decline, and the network energy almost runs out around 200 rounds, which is closely related to the high energy consumption transmission caused by its random cluster head mechanism. This further verifies its deficiency in energy utilization efficiency.

The PL-EBC, PSOE, and CGWOA algorithms perform better in energy control than LEACH. Their residual energy curves decline relatively gently, indicating that these algorithms have introduced energy perception or optimization mechanisms to some extent, reducing unnecessary energy waste. Among them, the energy consumption speed of PSOE is relatively fast in the early stage, while CGWOA still has a certain rapid energy decline phenomenon in the later stage, indicating that it still has room for improvement in global energy balance.

In contrast, the SHOE algorithm maintains the highest network residual energy throughout the simulation period, and its energy attenuation curve is the flattest. Even when the simulation terminates, the residual energy of SHOE outperforms other algorithms by a significant margin. This result indicates that SHOE effectively reduces long-distance high-energy consumption transmission through reasonable cluster head distribution and efficient routing strategies, significantly improving network energy utilization efficiency. In summary, the analysis of network residual energy further verifies the outstanding advantages of SHOE in energy efficiency and long-term network operation capability.

## 6. Conclusions and Future Prospects

This paper addressed the energy balance and lifecycle optimization issues of wireless sensor networks in scenarios with uneven node distribution, proposing the SHOE clustering routing algorithm that integrated the Spot Hyena Optimization Algorithm and the EEUC multi-hop relay mechanism. Through theoretical modeling and simulation experiments, it was verified that the SHOE algorithm innovatively used Gaussian distribution to achieve uneven node deployment, which was in line with the topological characteristics of “core dense, edge sparse” in actual monitoring scenarios [33,34], solving the problem of the insufficient adaptability of traditional algorithms. During the cluster head selection stage, by leveraging the hierarchical mechanism and dynamic parameter adjustment of SHOE’s α, β, δ, ω levels, it realized an adaptive trade-off between global exploration and local exploitation, which effectively prevented premature convergence combined with the empty cluster and isolated point repair mechanism, ensuring the robustness and rationality of the cluster structure. Inter-cluster communication selected the optimal transmission path through the energy difference criterion of EEUC multi-hop relay, decomposing long-distance high-energy consumption into short-distance low-energy consumption, significantly alleviating the “energy hotspot” problem. The simulation results showed that compared with four comparison algorithms (LEACH, PSOE, PL-EBC, CGWOA), the SHOE algorithm, after 2000 iterations, reduced the node death rate by more than 40% and increased the network’s remaining energy by more than 30%, and the volume of surviving nodes always led. The results sufficiently verified the distinct advantages of the SHOE algorithm regarding energy equilibrium, network stability, and prolonged network lifetime, thus supplying an efficient and applicable clustering routing approach for WSNs with uneven node distribution. Although the SHOE algorithm exhibited excellent performance in uneven node distribution scenarios, there was still room for further optimization and expansion. The current algorithm assumed node immobility. In the future, a mobile model (such as a random walk model) could be introduced to optimize the cluster head election cycle and routing update mechanism, thereby improving the algorithm’s adaptability to mobile WSN [35,36]. At the same time, the iterative optimization of SHOE still had certain computational overheads. In the future, by simplifying the fitness function and optimizing the iteration termination condition, the algorithm complexity could be reduced and could be adapted to resource-constrained sensor nodes. Additionally, the current algorithm focused on energy optimization as the core goal. In the future, multiple objective optimizations such as data transmission delay and link reliability could be introduced to meet the diverse needs of industrial monitoring and other scenarios [37,38,39,40,41].

## Figures and Tables

**Table 1:** Comparison table of the proposed algorithm with existing algorithms.

Method	Key Mechanism	Limitations	Improvement by SHOA
LEACH	Random CH election	Hotspot; unbalanced	Fitness-guided CH selection
PSOE	PSO optimizes intra-cluster distance	Ignores inter-cluster cost; hotspot	Global energy–distance joint optimization
PL-EBC	PSO + energy threshold + multi-hop	Single relay criterion; high complexity	Energy-difference Cost; dynamic relay
CGWOA	Chaotic GWO	Weak energy balance	EEUC multi-hop; structure repair
SHOE	SHO + improved EEUC + repair	—	Best balance and lifetime

**Table 2:** Core Energy Consumption Parameters Table.

Energy Consumption Type	Parameter Symbol	Value	Description
Transmit circuit energy consumption	ETX	50 × 10^−9^ J/bit	Fixed energy dissipation of the transceiver circuitry during data communication
Receive circuit energy consumption	ERX	50 × 10^−9^ J/bit	Fixed energy dissipation of the receiving circuit
Data aggregation energy consumption	EDA	5 × 10^−9^ J/bit	Energy dissipation of cluster heads during intra-cluster data aggregation
Short-range amplifier energy consumption	$E_{fs}$	10 × 10^−12^ J/bit/m^2^	Used when distance < d_0_ (free-space model)
Long-range amplifier energy consumption	$E_{mp}$	0.0013 × 10^−12^ J/bit/m^4^	Used when distance > d_0_ (multi-path fading model)
Distance threshold	$d_{0}$	d_0_ = $\sqrt{\frac{E_{fs}}{E_{mp}}}$ ≈ 87 m	Switching threshold for amplifier type

**Table 3:** List of all algorithm parameters.

Algorithm	Parameter Settings
LEACH	Cluster head probability P = 0.1
PSOE	Particle population = 30; inertia weight w = 0.7; acceleration coefficients c1 = c2 = 1.49
PL-EBC	Energy threshold factor α = 0.5; candidate cluster head ratio = 0.2
CGWOA	Convergence factor a ∈ [2, 0]; chaotic factor μ = 4
SHOE	Hyena population = 30; maximum iterations = 100; convergence factor A ∈ [2, 0]; empty cluster/isolation repair threshold = 50 m

## Data Availability

The data presented in this study are available on request from the corresponding author.
